# Headache in Cerebral Cavernous Malformations: Clinical, Genetic, and Neuroimaging Associations

**DOI:** 10.3390/jcm15187267

**Published:** 2026-09-18

**Authors:** Tamar Shiran, Daniel Rabinovitch, Shelly I. Shiran, Stephanie Libzon, Jonathan Roth, Shlomi Constantini, Amnon Mosek, Moran Hausman-Kedem

**Affiliations:** 1 Gray Faculty of Medical and Health Science, Tel Aviv University, Tel Aviv 6997801, Israel; tamarshir@tlvmc.gov.il (T.S.); shellyshi@tlvmc.gov.il (S.I.S.); jonathanr@tlvmc.gov.il (J.R.); shlomic@tlvmc.gov.il (S.C.); mosek.amnon@gmail.com (A.M.); 2 Department of Pediatrics, Dana-Dwek Children’s Hospital, Tel Aviv Sourasky University Medical Center, Tel Aviv 6423906, Israel; danielrab@tlvmc.gov.il; 3 Department of Radiology, Pediatric Radiology Unit, Dana-Dwek Children’s Hospital, Tel Aviv Sourasky University Medical Center, Tel Aviv 6423906, Israel; 4 Pediatric Neurology Institute, Dana-Dwek Children’s Hospital, Tel Aviv Sourasky University Medical Center, Tel Aviv 6423906, Israel; stephaniel@tlvmc.gov.il; 5 Department of Physical Therapy, School of Health Professions, Faculty of Medicine, Tel Aviv University, Tel Aviv 6997801, Israel; 6 Pediatric Neurosurgery Department, Tel Aviv Medical Center, Tel Aviv 6423906, Israel; 7 The Pediatric Brain Center, Dana-Dwek Children’s Hospital, Tel Aviv 6423906, Israel; 8 Department of Neurology, The Headache Clinic, Tel Aviv Medical Center, Tel Aviv Sourasky University Medical Center, Tel Aviv 6423906, Israel

**Keywords:** cerebral cavernous malformations, pediatrics, headache, migraine, neuroimaging, familial CCM

## Abstract

**Objectives**: Headache is reported in patients with cerebral cavernous malformations (CCMs), yet its prevalence and clinical associations remain poorly characterized. This study aimed to evaluate headache prevalence in children with CCMs and their affected adult relatives and to identify associated clinical, genetic, and neuroimaging associations. **Methods**: We conducted a cross-sectional study including pediatric patients with CCMs and their affected adult relatives. The occurrence of headache outside the context of acute CCM hemorrhage or surgery was assessed. Headache severity and disability were evaluated using the HIT-6 and MIDAS/pedMIDAS scales. **Results**: Of the 66 eligible patients, 63 were included in the final analysis (response rate 95.5%). Median age was 16.03 (range 3–66 years), and 29 (46%) had familial CCMs. Headaches were reported by 36 patients (57.1%), with tension-type headache being the most common phenotype (50.0%), followed by migraine (38.9%). Patients with headaches were older than those without headaches (median age 21 vs. 12.2 years, *p* = 0.008), and adults reported headaches more frequently than children (72.4% vs. 44.1%, *p* = 0.024). Patients with recurrent headaches were more likely to have presented with acute headache at the time of CCM diagnosis [26/32 (81.3%) vs. 8/18 (44.4%), *p* = 0.007; OR 5.42, 95% CI 1.8–19.6]. Headache occurrence was not associated with sex, genetic diagnosis, epilepsy, lesion number, or location. Hyperintense T1-weighted MRI signal within CCM lesions was associated with severe, disabling headache (MIDAS grade IV;6/12 [50%] vs. 0/7, *p* = 0.044). **Conclusions**: Headaches are common among patients with CCMs and increase with age regardless of lesion number and location. T1-weighted MRI hyperintensity within CCMs was associated with severe headache-related disability in adults. Headache in adult relatives may serve as a clinical clue to familial CCM carrier status. These findings require confirmation in larger studies.

## 1. Introduction

Cerebral cavernous malformations (CCMs) are low-flow vascular anomalies that affect 0.2 to 0.4% of the population [1]. Histologically, CCMs consist of clusters of dilated, endothelium-lined capillaries that lack intervening parenchyma, often leading to endothelial leakiness and intracranial hemorrhages (ICH) [2,3]. While often asymptomatic, acute or chronic ICH from CCMs may cause seizures or other focal neurological deficits [4,5]. On imaging, CCMs exhibit a characteristic “popcorn appearance” on T2-weighted magnetic resonance imaging (MRI) and blooming artifacts on susceptibility-weighted imaging (SWI) [1,6]. CCMs can occur sporadically or as a familial autosomal dominant condition linked to loss-of-function mutations in *KRIT1* (*CCM1*), *CCM2*, or *PDCD10* (*CCM3*) [7], which result in rho kinase (ROCK) activation [8]. Familial CCMs account for 10–40% of CCM cases and are classically characterized by multiple brain lesions and occasionally with lesions in the spine as well as extracerebral involvement [8,9]. Although CCMs are recognized as a plausible cause of secondary headaches in the International Classification of Headache Disorders (ICHD), diagnosis 6.3.4) [10], the prevalence of headache as well as its associations in patients with CCMs remain under-reported and poorly characterized, particularly in children. In a study of 126 symptomatic patients with *KRIT1*-related CCMs, only 4% presented with headaches [8]. In addition, isolated case reports suggest that some CCMs may trigger cluster headache-like, SUNCT-like, or migraine-like attacks [11]. The study aimed to investigate the prevalence and clinical, genetic, and radiological associations of headache in patients with CCM. Given the limited data on recurrent headache in CCM, we hypothesized that headache burden would be associated with age and specific clinical, genetic, and neuroimaging characteristics. We therefore aimed to characterize recurrent headache in children with CCM and their affected adult relatives, providing an integrated assessment of its prevalence, phenotype, and associations across clinical, genetic, and neuroimaging domains.

## 2. Methods

### 2.1. Study Design and Patient Population

This cross-sectional study was conducted at a tertiary referral pediatric medical center. The institutional review board approved the study (# 817-21). The primary objective was to evaluate the prevalence and burden of headaches in patients with CCMs. The secondary objective was to identify clinical, genetic, and neuroradiological associations with headache occurrence. Pediatric patients with CCMs diagnosed between 2007–2024, followed at a tertiary pediatric stroke and Cerebrovascular Disease Referral Center and/or the pediatric neurosurgery clinics during the study recruitment period (30 November 2022–31 December 2024) were identified. The study included children aged 3 to <18 years and their affected adult relatives with CCMs. Extensive family history of CCM was obtained during clinical visits. Affected adult relatives were defined as family members of pediatric patients, >18 years of age, with radiologically confirmed CCM on brain MRI. All known affected adult relatives were approached for study participation. Family members for whom more than one subject had CCM was diagnosed with familial CCM, regardless of genetic confirmation. Children younger than three years were excluded due to their limited ability to reliably recognize and report headache symptoms. Patients with other unrelated brain lesions that could potentially cause headaches were also excluded.

### 2.2. Data Collection

Demographic and clinical data were extracted from electronic records. Presenting symptoms, including headache, seizures, focal neurological deficits, as well as the indication for initial imaging, were recorded, along with age at symptom onset, or in asymptomatic patients, age at CCM diagnosis. History of symptomatic bleeding and prior surgeries for CCMs were also recorded. Data on CCM etiology were recorded, including positive family history/familial CCM, positive genetic diagnosis including the specific CCM gene involved, and radiation exposure. Familial CCM was defined by the presence of at least one family member with a confirmed CCM diagnosis. A positive genetic diagnosis was defined as the identification of a disease-related variant in a CCM-associated gene. Additional clinical data included a diagnosis of epilepsy or abnormal EEG findings, developmental delay, autism spectrum disorder (ASD), and abnormal neurological findings on examination. Family history of migraines was also recorded.

Brain imaging leading to the initial CCM diagnosis was performed according to routine clinical practice. Neuroradiological analyses to assess lesion characteristics were performed by a single neuroradiologist (SIS) blinded to clinical outcomes and headache status. MRI examinations were acquired on 1.5-T and 3.0-T scanners as part of routine clinical care. The sequences reviewed for neuroradiological assessment included axial susceptibility-weighted imaging (SWI), sagittal volumetric T1-weighted gradient-echo imaging, and axial T2-weighted turbo spin-echo (TSE) imaging. The following parameters were recorded based on the last follow-up MRI: CCM load (categorized as single, <5, 5–10, >10, or innumerable), lesion location (supratentorial, infratentorial, or both), and the presence of T1-weighted imaging (T1WI) hyperintensity in at least one CCM lesion.

### 2.3. Assessment of Headache

Participants or their legal caregivers provided informed consent and completed an anonymous questionnaire administered via an online link. Patients were considered to have headache if they reported recurrent episodes of headache occurring within the three months preceding study participation, outside episodes of acute bleeding or surgery. Initially, all patients were asked whether they experienced headache. Those reporting headaches subsequently completed the HIT-6 questionnaire [12], the MIDAS (for participants aged 18 and older) [13], or the PedMIDAS (for those under 18) [14] to assess headache severity and impact. Validated responses were collected and imported in their original form via the Qualtrics (Qualtrics, Provo, UT, USA) survey platform. Participants were categorized into headache and no-headache groups for comparative analyses.

Those reporting headache provided additional details through a clinical questioning performed by an experienced pediatric neurologist (MHK), including age at headache onset, characteristics of the headache, –quality of pain (throbbing, sharp, dull, pressure-like), location and laterality (unilateral/bilateral), duration, frequency, aggravating and mitigating factors and associated symptoms such as photophobia, phonophobia, nausea and vomiting, aura presence, and headache intensity as measured by the Visual Analogue Scale (VAS), and the use of analgesic for pain relief. Headaches were diagnosed and classified using completed questionnaires, medical records, and clinical interviews, based on the ICHD-3 criteria [10]. Headache phenotypes were classified according to ICHD-3 into the following headache disorder groups: migraine, tension-type headache, headache attributed to a substance or its withdrawal, and headache attributed to a cranial or cervical vascular disorder. Headache characterization and ICHD-3 classification were performed using a standardized, structured assessment based on patient-reported clinical features.

### 2.4. Statistical Analysis

Descriptive statistics were used to summarize the patients’ characteristics and to determine the prevalence of the primary and secondary outcomes. Continuous variables were summarized as median and interquartile range (IQR) and compared between groups using the Mann–Whitney U test. Categorical variables were presented as frequencies and percentages and compared using the chi-square test or Fisher’s exact test, as appropriate. Odds ratios (ORs) with 95% confidence intervals (CIs) were calculated for binary categorical comparisons. To account for potential confounding by age, multivariable logistic regression was performed with recurrent headache as the dependent variable and age entered as a continuous covariate. Results are reported as adjusted odds ratios (aORs) with 95% CIs. To account for multiple comparisons, *p*-values from the exploratory analyses were adjusted using the Benjamini–Hochberg false discovery rate (FDR) procedure. Both unadjusted and FDR-adjusted *p*-values are reported. FDR-adjusted *p*-values < 0.05 were considered statistically significant for analyses subject to multiple testing. All analyses were performed using IBM SPSS Statistics, version 29 (IBM Corp., Armonk, NY, USA). All tests were two-sided, and statistical significance was defined as *p* < 0.05.

#### Sample Size

An a priori sample size calculation was performed for the planned comparison between patients with and without recurrent headache. Assuming a moderate standardized between-group effect size (Cohen’s d = 0.5), 80% power, and a two-sided α of 0.05, approximately 64 participants were estimated to be required, assuming comparable group sizes.

## 3. Results

**Figure 1:**
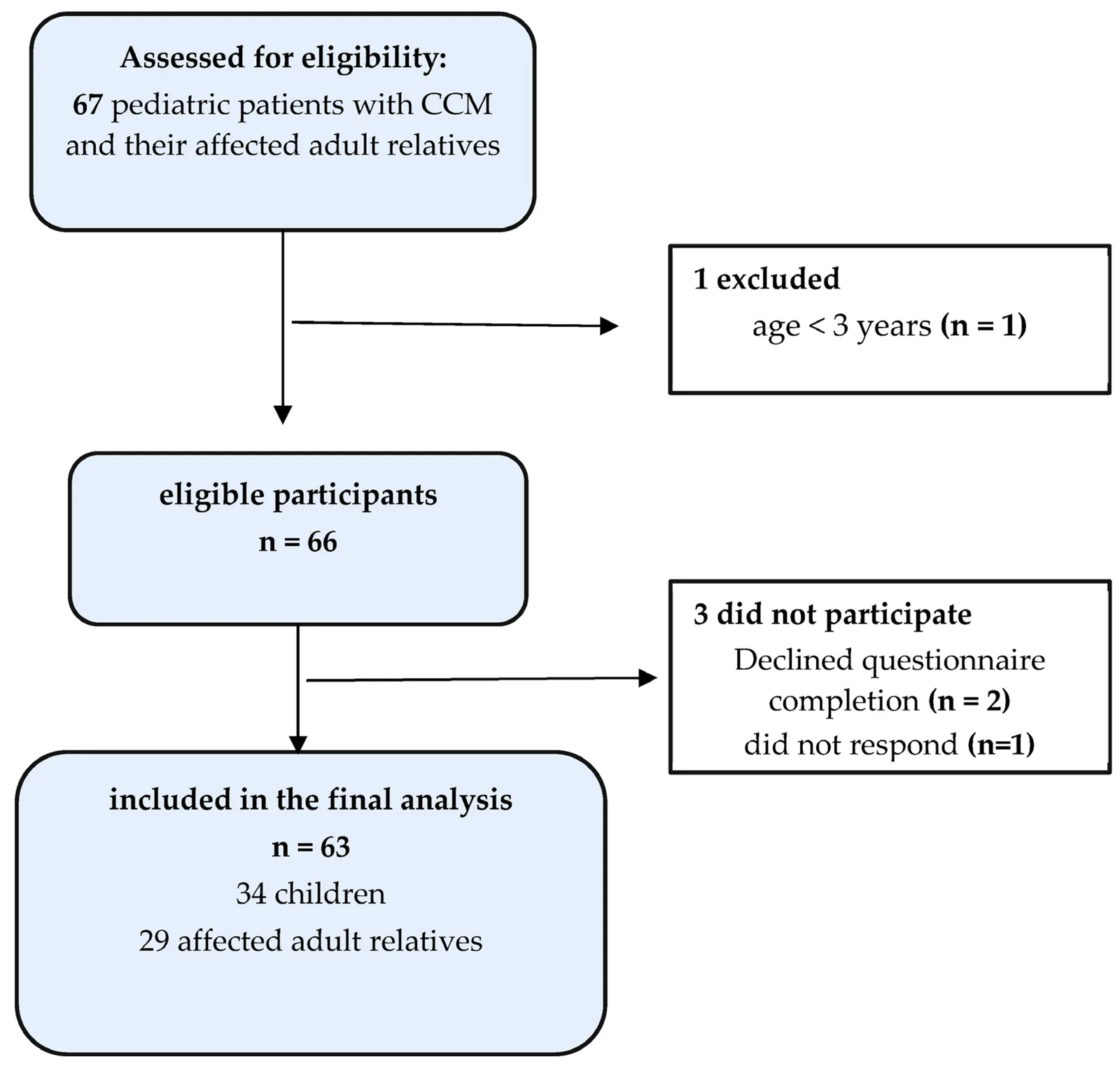
Study participant flow diagram, CCM, cerebral cavernous malformation.

Of the 67 individuals assessed for eligibility, one child was excluded due to age < 3 years. Of the remaining 66 eligible participants, 63 completed the study procedures and were included in the final analysis (response rate, 95.5%). Of the three eligible children who did not participate, two declined to complete the headache questionnaire, and one did not respond (Figure 1). The cohort had a median age of 16.03 years (range 3–66 years), included 33/63 (52.4%) males, and 34/63 (54%) children. Forty-five patients (71.4%) had multiple CCM lesions. Thirty-four patients (54.8%) had both supra- and infratentorial lesions, 23 (37.1%) had only supratentorial CCMs, and 5 (8.1%) had infratentorial lesion only. Familial CCM was present in 29/63 patients (46.0%). Genetic testing was performed in 46/63 patients (73.0%), of whom 35/46 (76.1%) had a positive genetic diagnosis (Table 1).

Most patients [50/63 (79.4%)] were symptomatic at presentation, with headache being the most common presenting symptom, reported in 34/50 patients (68%), followed by focal neurological deficits in 20/50 (40%) and seizures in 12/50 (24%). Headache as a presenting symptom was more frequent among patients with recurrent headache than among those without recurrent headache [26/32 (81.3%) vs. 8/18 (44.4%); OR 5.42, 95% CI 1.8–19.6; *p* = 0.007]. Recurrent headaches outside the acute hemorrhagic setting were reported by 36/63 participants (57.1%). Headache phenotypes are summarized in Table 2. The mean headache intensity on the VAS score was 6.1, and 3/36 (8.3%) patients reported headaches that awakened them from sleep. Analgesic use for pain relief was reported by 29 patients (80.6%).

Adults were significantly more likely to experience headaches than children [21/29, 72.4% vs. 15/34, 44.1%, *p* = 0.024, OR: 3.32, 95% CI: 1.15–9.59]. Similarly, the median age in the headache group was significantly higher than the non-headache group (21 vs. 12.2 years, *p* = 0.008). Patients with recurrent headaches outside the acute hemorrhagic setting were also more likely to present with headache at the time of CCM diagnosis compared with those without headaches (26/32, 81.3% vs 8/18, 44.4%, *p* = 0.007, OR: 5.417, 95% CI: 1.8–19.6). No significant difference was observed between groups with and without headache in terms of sex, age at diagnosis, genetic diagnosis, history of cranial radiation, history of symptomatic bleeding, prior CCM removal surgery, family history of migraine, or neurological comorbidities including epilepsy, ASD, or developmental delay. In addition, headache occurrence was not associated with CCM lesion number, location, or anatomical distribution. In an age-adjusted logistic regression model, headache as the presenting symptom remained independently associated with recurrent headache (aOR 4.49, 95% CI 1.19–16.92; *p* = 0.026).

**Figure 2:**
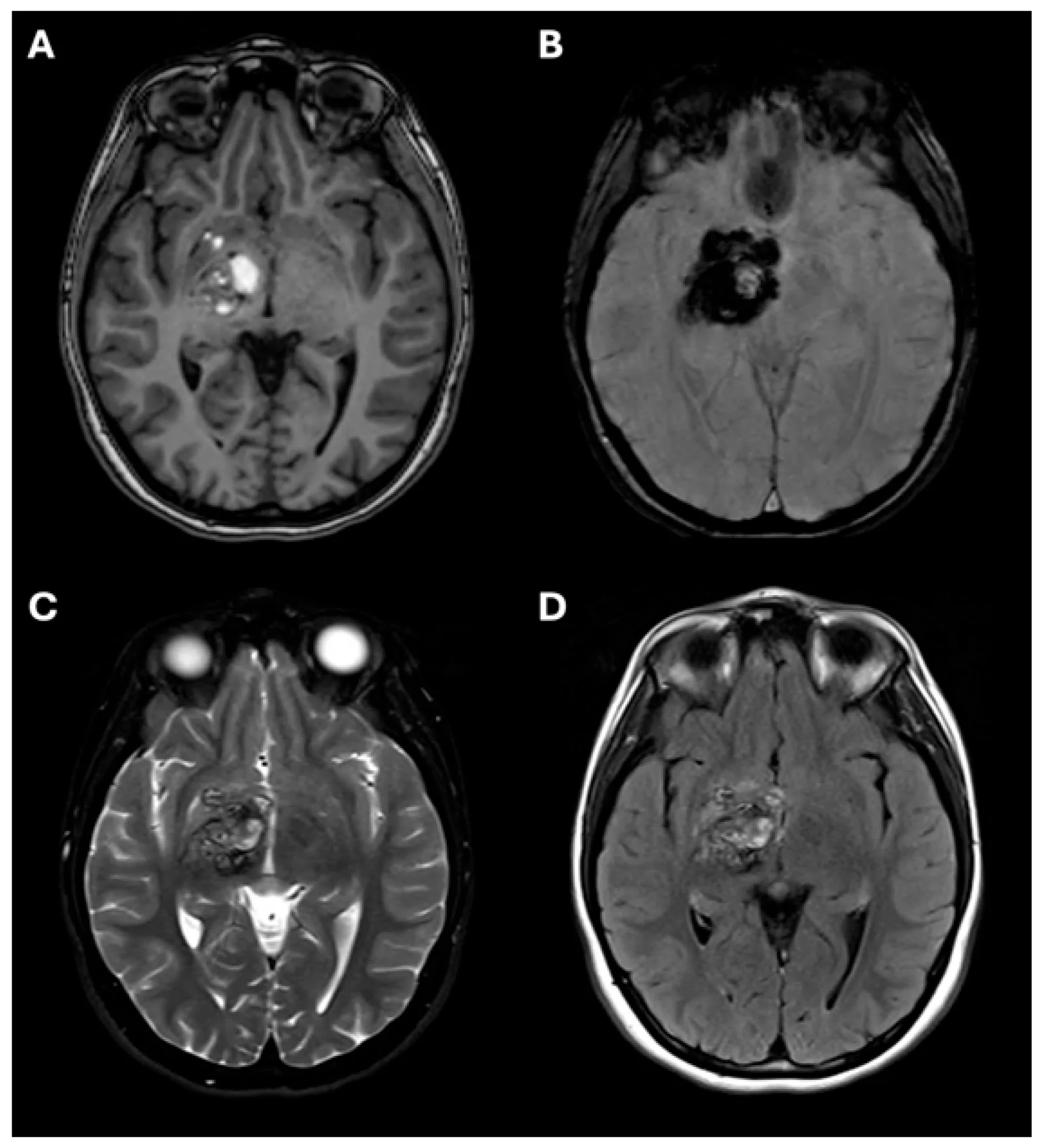
Brain MRI of a 13.5-year-old female with cerebral cavernous malformations (CCMs). Axial (**A**) T1-weighted image demonstrates intrinsic T1 hyperintensity; (**B**) susceptibility-weighted imaging (SWI) demonstrates prominent blooming artifact; and (**C**) T2-weighted and (**D**) T2-weighted FLAIR images demonstrate the characteristic heterogeneous “popcorn” appearance of CCMs.

T1WI hyperintensity within at least one CCM lesion was identified in 31/62 patients (50%) (Figure 2). Among the 19 adult participants assessed using MIDAS, grade IV headache-related disability was observed in 6/12 (50%) patients with T1WI hyperintensity compared with 0/7 patients without T1WI hyperintensity (Fisher’s exact test, *p* = 0.044).

## 4. Discussion

Data on the prevalence, characteristics, and burden of headaches in patients with cerebral cavernous malformations (CCMs) remain limited, particularly in children and outside the setting of acute hemorrhage. In this study, we evaluated headache burden in a pediatric CCM cohort and their affected adult relatives and provide updated insights into associated clinical, genetic, and neuroimaging correlates.

Headache was reported by 57.1% of the overall cohort and was more prevalent among affected adult relatives than among children with CCMs (72.4% vs. 44.1%). For context, a large population-based review reported headache prevalence of about 46% in adults and 51% in children in the general population [15]. However, differences in headache definitions and assessment time frames limit direct comparison with our cohort.

Recurrent headaches were reported more frequently in affected adult relatives than in children with CCMs. This difference may partly reflect younger children’s limited ability to recognize or reliably report headache symptoms. However, our study included only verbal children aged ≥ 3 years of age, thereby trying to minimize this potential bias. Alternatively, headaches may become more apparent with age as CCM lesions undergo repeated microhemorrhages and progressive hemosiderin deposition, processes that may increase local inflammatory activity and pain sensitivity over time. Importantly, the diagnosis of a child with multiple CCMs often raises suspicion for familial CCM, a condition typically inherited in an autosomal dominant pattern. In this context, headaches in a parent of a child with CCM may potentially serve as a useful clinical clue prompting further genetic or neuroradiological evaluation, potentially facilitating the earlier identification of previously undiagnosed affected family members.

In our study, headache was the most common presenting symptom at CCM diagnosis, occurring in 68% of symptomatic patients. The prevalence of headache as a presenting symptom of CCMs reported in other studies ranges from 6% to 65%, depending on the study population and diagnostic criteria used to define symptomatic presentation [16]. Similar findings were reported by Jaman et al. [17], who also identified headaches as the most common initial symptom in pediatric patients, occurring in 32% of their cohort. In contrast, headache was reported as the presenting symptom in only 4% of patients in a previous cohort of symptomatic *KRIT1*-related CCMs [8]. This marked difference may reflect differences in study populations and ascertainment between studies. The higher rates of acute headache at presentation in our study can potentially result from the high proportion of children included in our study, in whom the threshold for performing MRI as part of the diagnostic workup of headache is high compared to adults. In addition, recruitment from a tertiary pediatric vascular neurology and neurosurgery referral center may have introduced ascertainment bias by enriching the cohort for symptomatic patients. Notably, in our cohort, headache as a presenting symptom was more frequent among patients with recurrent headache than among those without recurrent headache (81.3% vs. 44.4%). However, this association should be interpreted cautiously, as it may partly reflect an incorporation effect, since patients whose CCM was initially identified during evaluation for headache are inherently more likely to report recurrent headache.

We found no significant association between recurrent headaches and CCM lesion number, lesion location, positive genetic diagnosis, or T1WI hyperintensity. These findings suggest that headache in CCMs may not be fully explained by anatomic factors such as lesion burden or distribution. Instead, headache may also reflect underlying biological processes including local inflammatory activity within the lesions’ microvascular milieu [18,19,20]. Although T1WI hyperintensity was not associated with recurrent headaches in the overall cohort, it was associated with grade IV headache-related disability among adults assessed with MIDAS. In contrast, no corresponding association was observed among pediatric participants assessed with PedMIDAS. T1WI hyperintensity typically reflects the presence of extracellular or intracellular methemoglobin and thrombosis, suggesting recent or subacute blood degradation products within the lesion. Repeated microhemorrhages may irritate adjacent brain tissue or meninges and trigger local inflammatory responses, which could contribute to headache generation. In addition, subacute hemorrhage may produce mild mass effect or local tissue irritation that may further exacerbate headache severity [21,22]. These mechanisms provide a possible biological context for the observed association between T1WI hyperintensity and headache-related disability in adults. However, given the small number of participants with MIDAS grade IV disability, the absence of a corresponding association in the PedMIDAS group, and the lack of direct assessment of these proposed mechanisms, this observation should be considered hypothesis-generating and requires confirmation in larger studies.

The management of headaches in patients with CCM remains insufficiently studied. The safety of commonly used acute migraine therapies, such as triptans, has not been systematically evaluated in this population, and treatment decisions therefore rely largely on clinical judgment. Among the preventive therapies, propranolol, which is widely used for migraine prophylaxis, has been suggested in experimental and clinical observations to exert stabilizing effects on vascular malformations and may potentially reduce CCM lesion activity [23,24]. However, these studies were not designed to evaluate its efficacy for headache prevention in patients with CCM and therefore do not support specific therapeutic recommendations for headache management in this population. Propranolol may represent a potential preventive option for patients with CCM who experience recurrent headaches. Further prospective studies are needed to clarify optimal treatment strategies for headache management in patients with CCM.

This study has several limitations. First, its cross-sectional, single-center design precludes causal inference and limits the precision of the estimates. Several potential biases are plausible, including selection and ascertainment biases secondary to patients’ recruitment through a tertiary pediatric referral center, as well as recall and reporting biases. Another limitation relates to the attribution of headaches specifically to CCM. Given the high prevalence of headache disorders in the general population [15] and the absence of an unaffected control group, headaches in our cohort may represent coincidental primary headache disorders, rather than CCM-related headaches. Future controlled studies are needed to clarify this association. In addition, as MRI examinations were obtained as part of routine clinical care, the interval between MRI and headache assessment was not controlled and was reviewed by a single neuroradiologist without formal assessment of reproducibility. Associated developmental venous anomalies and additional markers of lesion activity, including advanced imaging techniques capable of quantitatively assessing vascular hyperpermeability and iron deposition at the lesion site and interval changes on serial imaging, were not systematically performed. Despite these limitations, this study uniquely integrates clinical, genetic, and imaging data across both pediatric and adult patients, therefore offering a comprehensive, real-life analysis of headache burden in CCMs, a perspective rarely addressed in previous literature.

In conclusion, headaches outside the acute hemorrhage setting are common and potentially disabling in children with CCM and their affected adult relatives, with a tendency to increase with age. Headache may therefore represent an important clinical clue to yet-undiagnosed affected adult family members in familial CCM cases. Importantly, headaches may reflect underlying vascular or neuroinflammatory processes, warranting further investigation into potential pathophysiological mechanisms. The observed association between T1-weighted MRI hyperintensity and severe headache-related disability in adults should be considered exploratory and hypothesis-generating and requires confirmation in larger studies. Improved recognition of headache burden in CCM may support more comprehensive clinical assessment and management of headache in this population.

## Figures and Tables

**Table 1:** Characteristics of the study population.

	All Patients (n = 63)	No Headache (n = 27)	Headache (n = 36)	95% OR (CI)	*p*-Value	FDR-Adjusted *p*-Value
Age (years), median (IQR)	16.03 (11.6, 32.9)	12.2 (9, 20.7)	21 (14, 35.7)		**0.008**	**0.024**
Male (%)	33 (52.4%)	17 (63%)	16 (44.4%)	2.125 (0.77–5.9)	0.145	0.145
Adults (%)	29 (46%)	8 (29.6%)	21 (58.3%)	**3.32 (1.15–9.59)**	**0.024**	**0.036**
Familial CCMs	29 (46% (	13 (48.1%)	16 (44.4%)	0.82 (0.19–3.54)	1	1
**Genetic characteristics**						
Positive genetic diagnosis (CCM genes) (n = 46)	35/46 (76.1%)	16 (72.7%)	19 (79.2%)	1.425 (0.37–5.55)	0.609	1
*CCM1*	9 (24.3%)	6 (35.3%)	3 (15%)	0.34 (0.07–1.68)	0.255	0.638
*CCM2*	16 (43.2%)	5 (29.4%)	11 (55%)	2.9 (0.75–11.49)	0.117	0.585
*CCM3*	8 (21.6%)	4 (23.5%)	4 (20%)	0.81 (0.17–3.9)	1	1
Other genes	2 (5.4%)	1 (5.9%)	1 (5%)	0.84 (0.05–14.56)	1	1
**Indication for first imaging**						
Incidental	5 (7.9%)	4 (14.8%)	1 (2.8%)	0.16 (0.02–1.56)	0.155	0.31
Positive family history	15 (23.8%)	5 (18.5%)	10 (27.8%)	1.69 (0.5–5.7)	0.393	0.393
Symptomatic (n = 50)	50 (79.4%)	18 (66.7%)	32 (88.9%)	**4 (1.08–14.85)**	**0.031**	0.093
Presenting symptom among symptomatic patients—headache	34/50 (68%)	8/18 (44.4%)	26/32 (81.3%)	**5.42 (1.8–19.6)**	**0.007**	**0.042**
Presenting symptom among symptomatic patients—epilepsy	12/50 (24%)	6/18 (33.3%)	6/32 (18.8%)	0.46 (0.12–1.73)	0.309	0.3708
Presenting symptom among symptomatic patients—other	20/50 (40%)	9/18 (50%)	11/32 (34.4%)	0.52 (0.16–1.70)	0.279	0.3708
Age at diagnosis of CCM (years), median (IQR)	11 (5.3, 21.1)	9.2 (4.6, 13)	13.8 (7.2, 25.5)		0.073	0.365
History of prior neurosurgical resection of CCM	14 (22.2%)	5 (18.5%)	9 (25%)	1.4 (0.41–4.8)	0.592	0.74
Family history of migraine	33 (52.4%)	13 (48.1%)	20 (55.6%)	1.33 (0.48–3.69)	0.580	0.74
Abnormal neurological examination	17 (27%)	7 (25.9%)	10 (27.8%)	1.12 (0.35–3.52)	0.849	0.849
**Neurological comorbidities**						
Epilepsy	5 (7.9%)	2 (7.4%)	3 (8.3%)	1.14 (0.18–7.32)	1	1
Developmental delay	11 (17.5%)	5 (18.5%)	6 (16.7%)	0.88 (0.24–3.25)	1	1
ASD	5 (7.9%)	3 (11.1%)	2 (5.6%)	0.47 (0.07–3.035)	0.643	1
Abnormal EEG findings (n = 33)	17/33 (51.5%)	8/15 (53.3%)	9/18 (50.0%)	0.88 (0.22–3.45)	1	1
**Radiological characteristics**						
**CCMs number**					0.811	0.811
Single	18 (28.6%)	6 (22%)	12 (33.3%)			
<5	15 (23.8%)	7 (25.9%)	8 (22.2%)			
5–10	11 (17.5%)	6 (22.2%)	5 (13.9%)			
>10	15 (23.8%)	6 (22.2%)	9 (25%)			
Multiple (innumerable)	4 (6.3%)	2 (7.4%)	2 (5.6%)			
**CCMs location** (n = 62)					0.162	0.486
Supratentorial	23/62 (37.1%)	10/26 (38.5%)	13/36 (36.1%)			
Infratentorial	5/62 (8.1%)	5/62 (8.1%)	5/62 (8.1%)			
Supratentorial and Infratentorial	34/62 (54.8%)	16/26 (61.5%)	18/36 (50%)			
T1WI hyperintensity in at least one CCM (n = 62)	31/62 (50.0%)	14/26 (53.8%)	17/36 (47.2%)	0.77 (0.28–2.11)	0.607	0.811

Abbreviations: CCM = cerebral cavernous malformation; IQR = interquartile range; EEG = electroencephalography; ASD = autism spectrum disorder, T1WI = T1-weighted imaging. For each variable, n indicates the total number of participants with available data. Subgroup-specific denominators are shown as n/N (%) where applicable.

**Table 2:** Headache phenotypes according to ICHD-3 classification.

ICHD-3 Headache Disorder Group	Specific ICHD-3 Diagnosis	n
Migraine	Chronic migraine	3
	Migraine without aura	5
	Migraine with aura	1
	Probable migraine	5
Tension-type headache	Episodic tension-type headache	14
	Chronic tension-type headache	4
Headache attributed to a substance or its withdrawal	Medication-overuse headache	1
Headache attributed to cranial or cervical vascular disorder	Possible headache attributed to cavernous angioma *	3
Total		36

**Abbreviations**: CCM, cerebral cavernous malformation; ICHD-3, International Classification of Headache Disorders, 3rd edition. * Possible headache attributed to cavernous angioma was assigned when the clinical presentation suggested a secondary CCM-related headache, but full ICHD-3 causation criteria could not be established.

## Data Availability

The data presented in this study are available on request from the corresponding author. The data are not publicly available due to privacy and ethical restrictions.

## References

[1] Al-Shahi Salman R., Berg M.J., Morrison L., Awad I.A., Angioma Alliance Scientific Advisory Board (2008). Hemorrhage from cavernous malformations of the brain: Definition and reporting standards. Angioma Alliance Scientific Advisory Board. Stroke.

[2] Robinson J.R., Awad I.A., Masaryk T.J., Estes M.L. (1993). Pathological heterogeneity of angiographically occult vascular malformations of the brain. Neurosurgery.

[3] Tanriover G., Sozen B., Seker A., Kilic T., Gunel M., Demir N. (2013). Ultrastructural analysis of vascular features in cerebral cavernous malformations. Clin. Neurol. Neurosurg..

[4] Labauge P., Denier C., Bergametti F., Tournier-Lasserve E. (2007). Genetics of cavernous angiomas. Lancet Neurol..

[5] Morris Z., Whiteley W.N., Longstreth W.T., Weber F., Lee Y.-C., Tsushima Y., Alphs H., Ladd S.C., Warlow C., Wardlaw J.M. (2009). Incidental findings on brain magnetic resonance imaging: Systematic review and meta-analysis. BMJ.

[6] Gomez-Paz S., Maragkos G.A., Salem M.M., Ascanio L.C., Lee M., Enriquez-Marulanda A., Orrego-Gonzalez E., Kicielinski K., Moore J.M., Ogilvy C.S. (2020). Symptomatic Hemorrhage From Cerebral Cavernous Malformations: Evidence from a Cohort Study. World Neurosurg..

[7] Zafar A., Quadri S.A., Farooqui M., Ikram A., Robinson M., Hart B.L., Mabray M.C., Vigil C., Tang A.T., Kahn M.L. (2019). Familial cerebral cavernous malformations. Stroke.

[8] Denier C., Labauge P., Brunereau L., Cavé-Riant F., Marchelli F., Arnoult M., Cecillon M., Maciazek J., Joutel A., Tournier-Lasserve E. (2004). Clinical features of cerebral cavernous malformations patients with KRIT1 mutations. Ann. Neurol..

[9] Rigamonti D., Hadley M.N., Drayer B.P., Johnson P.C., Hoenig-Rigamonti K., Knight J.T., Spetzler R.F. (1988). Cerebral cavernous malformations. Incidence and familial occurrence. N. Engl. J. Med..

[10] ICHD-3 (2018). The International Classification of Headache Disorders, 3rd edition. Cephalalgia.

[11] Favier I., van Vliet J.A., Roon K.I., Witteveen R.J.W., Verschuuren J.J.G.M., Ferrari M.D., Haan J. (2007). Trigeminal autonomic cephalgias due to structural lesions: A review of 31 cases. Arch. Neurol..

[12] Yang M., Rendas-Baum R., Varon S.F., Kosinski M. (2011). Validation of the Headache Impact Test (HIT-6TM) across episodic and chronic migraine. Cephalalgia.

[13] Stewart W.F., Lipton R.B., Kolodner K.B., Sawyer J., Lee C., Liberman J.N. (2000). Validity of the Migraine Disability Assessment (MIDAS) score in comparison to a diary-based measure in a population sample of migraine sufferers. Pain.

[14] Hershey A.D., Powers S.W., Vockell A.L., LeCates S., Kabbouche M.A., Maynard M.K. (2001). PedMIDAS: Development of a questionnaire to assess disability of migraines in children. Neurology.

[15] Stovner L., Hagen K., Jensen R., Katsarava Z., Lipton R., Scher A., Steiner T.J., Zwart J.-A. (2007). The global burden of headache: A documentation of headache prevalence and disability worldwide. Cephalalgia.

[16] Washington C.W., McCoy K.E., Zipfel G.J. (2010). Update on the natural history of cavernous malformations and factors predicting aggressive clinical presentation. Neurosurg. Focus.

[17] Jaman E., Abdallah H.M., Zhang X., Greene S. (2023). Clinical characteristics of familial and sporadic pediatric cerebral cavernous malformations and outcomes. J. Neurosurg. Pediatr..

[18] Retta S.F., Glading A.J. (2016). Oxidative stress and inflammation in cerebral cavernous malformation disease pathogenesis: Two sides of the same coin. Int. J. Biochem. Cell Biol..

[19] Awad I.A., Polster S.P. (2019). Cavernous angiomas: Deconstructing a neurosurgical disease. J. Neurosurg..

[20] Bianconi A., Salvati L.F., Perrelli A., Ferraris C., Massara A., Minardi M., Aruta G., Rosso M., Massa Micon B., Garbossa D. (2022). Distant Recurrence of a Cerebral Cavernous Malformation in the Vicinity of a Developmental Venous Anomaly: Case Report of Local Oxy-Inflammatory Events. Int. J. Mol. Sci..

[21] Zimny A., Zińska L., Bladowska J., Neska-Matuszewska M., Sąsiadek M. (2013). Intracranial lesions with high signal intensity on T1-weighted MR images—Review of pathologies. Pol. J. Radiol..

[22] Ginat D.T., Meyers S.P. (2012). Intracranial lesions with high signal intensity on T1-weighted MR images: Differential diagnosis. Radiographics.

[23] Lanfranconi S., Scola E., Meessen J.M.T.A., Pallini R., Bertani G.A., Al-Shahi Salman R., Dejana E., Latini R., Abete Fornara G., Treat_CCM Investigators (2023). Safety and efficacy of propranolol for treatment of familial cerebral cavernous malformations (Treat_CCM): A randomised, open-label, blinded-endpoint, phase 2 pilot trial. Lancet Neurol..

[24] Ikramuddin S., Liu S., Ryan D., Hassani S., Hasan D., Feng W. (2024). Propranolol or Beta-Blockers for Cerebral Cavernous Malformation: A Systematic Review and Meta-analysis of Literature in Both Preclinical and Clinical Studies. Transl. Stroke Res..

